# Understanding how an amphicarpic species with a mixed mating system responds to fire: a population genetic approach

**DOI:** 10.1093/aobpla/plab067

**Published:** 2021-10-21

**Authors:** Elena M Meyer, Joel F Swift, Burgund Bassüner, Stacy A Smith, Eric S Menges, Brad Oberle, Christine E Edwards

**Affiliations:** 1 Center for Conservation and Sustainable Development, Missouri Botanical Garden, 4344 Shaw Blvd., St. Louis, MO 63110, USA; 2 Division of Natural Sciences, New College of Florida, 5800 Bay Shore Road, Sarasota, FL 34243, USA; 3 Plant Ecology Program, Archbold Biological Station, 123 Main Drive, Venus, FL 33960, USA

**Keywords:** Amphicarpy, chasmogamy, cleistogamy, fire ecology, genetic structure, heterozygosity, inbreeding, mixed mating systems, self-pollination

## Abstract

Amphicarpic plants produce both above-ground and below-ground seeds. Because below-ground seeds are protected in the soil and may maintain viability when above-ground conditions are stressful, they were proposed as an adaptation to recolonize a site after disturbance. However, whether below-ground seeds are the main colonizers after a disturbance remains unknown. Our goal was to understand whether recolonization by an amphicarpic species after fire was accomplished primarily through germination of seeds produced above-ground or below-ground. We investigated *Polygala lewtonii*, an amphicarpic, perennial species endemic to fire-prone Florida sandhill and scrub, where fire kills plants but subsequently increases recruitment and population sizes. *Polygala lewtonii* produces three flower types: above-ground chasmogamous flowers and above-ground and below-ground cleistogamous flowers, with previous research demonstrating chasmogamous flowers produce a much greater proportion of seeds than cleistogamous flowers. We quantified outcrossing in seeds produced by chasmogamous flowers to determine whether it differed from the 100 % self-fertilized below-ground seeds. Approximately 25 % of seeds from chasmogamous flowers showed evidence of cross-pollination. Assuming that chasmogamous flowers produce the majority of the above-ground seeds, as was shown previously, this indicates it is possible to differentiate between germination by above-ground versus below-ground seeds in post-fire colonization. We next compared genetic diversity, admixture, inbreeding and population genetic structure pre- and post-fire. If fire promoted germination of chasmogamous seeds, heterozygosity and admixture would increase, and genetic structure and inbreeding would decrease. Instead, inbreeding and genetic structure increased and admixture decreased, suggesting that the below-ground selfed seeds (with limited dispersal ability) increased their contribution to the population after fire, possibly because fire reduced above-ground seed viability. Additionally, new alleles not found previously in range-wide analyses emerged from the seed bank post-fire. These results suggest that amphicarpy is a powerful adaptation to preserve genetic variation, maintain adaptive potential and promote rapid post-fire colonization.

## Teaser

We examined the mating system’s response to fire in *Polygala lewtonii*, a species endemic to fire-prone Florida sandhill and scrub. *Polygala lewtonii* has three flower types: above-ground chasmogamous (open-pollinated) and cleistogamous (closed, obligately self-fertilizing) flowers and below-ground cleistogamous flowers, with fruits produced both above-ground and below-ground. We used a genetic approach to test whether post-fire recolonization was accomplished primarily through germination of above-ground or below-ground seed. The below-ground selfed seeds showed an increased contribution to the population and new alleles emerged from the seed bank post-fire. These results suggest that amphicarpy is an adaptation to promote rapid post-fire colonization and preserve genetic variation and adaptive potential.

## Introduction

Understanding how reproductive strategies evolve in disturbance-maintained ecosystems connects basic evolutionary theory to conservation applications. Episodic mortality from regular disturbance tends to reduce population genetic variation and increase inbreeding (Dolan *et al.* 2008; Davies *et al.* 2016) while selecting for life history traits that maintain reproductive potential (Lytle 2001). Adaptive reproductive strategies may promote reproductive assurance while constraining genetic variation and dispersal to the point of increasing extinction risk (Goldberg and Igić 2012; Vamosi *et al.* 2018). However, seed dormancy and banking in the soil may provide a reservoir for genetic diversity (Vandvik *et al.* 2016). Effective conservation management of species endemic to disturbance-maintained ecosystems requires special attention to connections between disturbance, demography, mating system and genetic diversity.

A relatively common reproductive strategy among plant species in disturbance-prone habitats is mixed mating. Mixed mating is characterized by both self- and cross-fertilization and a proportion of selfing between 0.2 < *t* ≤ 0.8 (Goodwillie *et al.* 2005). Mixed mating systems are hypothesized to be an adaptation to ecosystems that regularly experience disturbance, providing reproductive assurance through self-fertilization when pollinators or conspecifics are absent (Cheplick 1987; Symonides 1988). However, reliance on self-fertilization may lead to inbreeding depression (i.e. decreased fitness of individuals from increased homozygosity for recessive deleterious alleles), although purging of deleterious alleles through selection may mitigate this effect in heavily selfing lineages (Holsinger 1988).

A second, less common life history trait proposed to be an adaptation to frequent disturbance is amphicarpy, a form of dimorphism where both aerial and subterranean flowers and seeds are produced on the same individual (Cheplick 1987). Amphicarpic plants often exhibit seed polymorphism (Symonides 1988), with below-ground, obligately selfing or cleistogamous (CL) flowers producing larger seeds with limited dispersal potential, and above-ground, open-pollinated or chasmogamous (CH) flowers producing smaller seeds suitable for dispersal across larger distances (Cheplick 1987; Kaul *et al.* 2000; Zhang *et al.* 2020). Below-ground seeds may be an adaptation to disturbance-prone habitats; they remain below the soil surface, are protected from disturbances such as fire and may be able to quickly recolonize a site, provided that they maintain viability and dormancy until stimulated by disturbance (Cheplick and Quinn 1987, 1988). Although previous studies of one amphicarpic annual species found that seeds buried below-ground showed greater germination than surface-sown seeds after a fire (Cheplick and Quinn 1987, 1988), to our knowledge, no study has quantified whether seeds produced by below-ground CL flowers of amphicarpic species are the predominant mechanism to recolonize sites after a disturbance.

*Polygala lewtonii* (Polygalaceae) is a federally endangered (USFWS 1999), amphicarpic perennial plant species endemic to fire-prone Florida sandhill and scrub. Previous studies found that *P. lewtonii* is adapted to fire; although plants are killed by fire and they do not re-sprout after fire except in rare cases (i.e. very low-intensity fire), smoke stimulated seed germination (Lindon and Menges 2008) and population sizes increased dramatically after burning (Weekley and Menges 2012). *Polygala lewtonii* is one of few species exhibiting mixed mating and amphicarpy via three types of flowers: (i) CH flowers, (ii) above-ground CL flowers and (iii) below-ground CL flowers. In *P. lewtonii*, open-pollinated CH flowers also have a delayed selfing mechanism (Weekley and Brothers 2006), which was proposed to provide reproductive assurance in the absence of pollinators (Cheplick 1987; Symonides 1988). Although *P. lewtonii* matures both above-ground and below-ground fruits, flowers vary in abundance and rates of fruit production, with CH flowers producing a median of 26 fruits per plant, above-ground CL flowers producing 2 fruits per plant and below-ground CL flowers producing 1.5 fruits per plant (Koontz *et al.* 2017). Thus, CH flowers produced over seven times the number of fruits produced by above- and below-ground CL flowers combined (Koontz *et al.* 2017). All seeds produce an elaiosome, a fleshy appendage that attracts ants for seed dispersal (Zomlefer 1989). Ants collect the above-ground seeds (USFWS 1999; Weekley and Menges 2003) and may disperse them up to several meters. Ants do not access nor disperse the below-ground CL seeds (Weekley and Brothers 2006), meaning below-ground dispersal is likely limited to the length of the rhizomes (<1 m).

Although *P. lewtonii* was the subject of a previous fine-scale population genetic analysis to understand its predominant mating system and patterns of population genetic structure (Swift *et al.* 2016), it is unknown how these characteristics are affected by fire. Genetic results indicated that reproduction in *P. lewtonii* occurred predominantly via inbreeding, outcrossing rates were low and genetic variation was structured at a very fine scale (Swift *et al.* 2016). The low outcrossing rate was unexpected because the showy, open-pollinated CH flowers produce most seeds in *P. lewtonii* (Koontz *et al.* 2017), and pollinator exclusion reduced seed set by almost 90 % (Weekley and Brothers 2006). This indicates that only a small proportion of fruits were produced via delayed selfing. Swift *et al.* (2016) hypothesized that fire may be necessary to stimulate germination of above-ground seed, and that the low outcrossing rate may be due to a lack of recent fire. If fire stimulates the germination of above-ground seed, we would expect increased heterozygosity and decreased inbreeding and genetic structure after a fire, assuming both that CH flowers produce the majority of above-ground seed, as was shown previously (Koontz *et al.* 2017), and that a moderate proportion of those seeds are produced via outcrossing. Conversely, other authors suggested that fire may decrease the survival rates of above-ground seeds and seedlings, such that below-ground CL flowers and seeds of amphicarpic species may predominantly recolonize a site after a disturbance (Cheplick and Quinn 1987, 1988). In this case, because below-ground CL seeds are 100 % selfed and can disperse only the length of the rhizome, we would expect increased inbreeding and genetic structure after a fire.

We utilized a population genetic approach to analyse the dynamics of recolonization of the amphicarpic species *P. lewtonii* after fire. Our analyses were two-pronged. We first tested whether we could differentiate among seed types in post-fire recolonization. We quantified the outcrossing rate in seeds produced by CH flowers by comparing their genotypes to those of their maternal parents; we then tested whether they deviated from the patterns expected for 100 % selfing of below-ground CL seeds. Because CH flowers exhibited low fruit set when pollinators were limited (Weekley and Brothers 2006), we hypothesized that most CH flowers would be the product of outcrossing. Specifically, we expected to encounter non-maternal alleles that could not be the product of selfing in a majority of seeds and heterozygosity that significantly exceeded the 50 % reduction expected for a single generation of pure selfing.

Second, we compared the genetic diversity and structure of populations before and after fire. We tested how heterozygosity, inbreeding coefficients, genetic diversity, number of private alleles and genetic structure changed after a prescribed burn in a natural population of *P. lewtonii*. If burning stimulated germination of seed from above-ground flowers, then we would expect increased heterozygosity and admixture and decreased inbreeding and spatial population genetic structure post-fire. This assumes that CH flowers produce a moderate proportion of seed through outcrossing and a much greater proportion of seed than above-ground CL flowers (Koontz *et al.* 2017). Conversely, if burning reduced the viability of above-ground seeds and stimulated germination of seed produced by below-ground flowers, which are obligately selfing and disperse >1 m, then we would expect to find decreased heterozygosity and admixture and increased inbreeding and among-group spatial population genetic structure post-fire. The results of these experiments have important consequences for the conservation of *P. lewtonii* and more broadly for our understanding of the function of amphicarpy.

## Materials and Methods

### Study species

*Polygala lewtonii* (Polygalaceae) is a short-lived (2–10 years) perennial, reaching ~20 cm tall (Weekley and Brothers 2006). The plant bears clusters of leaves that overlap on the glabrous stem like shingles (Small 1933; USFWS 1999). Chasmogamous flowers are deep pink to purple, occur on densely flowered terminal racemes (Weekley and Brothers 2006) and are visited by a variety of insect species, including sulfur butterflies and bee flies (Weekley and Brothers 2006). Rarely, solitary, pale pink above-ground CL flowers occur in the axils of the above-ground leaves on short leafless branches (Weekley and Brothers 2006; Wunderlin *et al.* 2021). Below-ground, white CL flowers occur sparsely on the rhizomes. Given that the *P. lewtonii* shows no evidence of clonal reproduction and only rarely re-sprouts after fire, the primary function of the rhizomes is to promote sexual reproduction by bearing the below-ground CL flowers and facilitating the dispersal of seeds produced by below-ground CL flowers. *Polygala lewtonii* flowers throughout the year, with CH flowers present from January to May, aerial CL flowers present from June to January and below-ground CL flowers present from July to February (Koontz *et al.* 2017). Fruits are oblong, ca. 3–4 mm long (Wunderlin *et al.* 2021), dehiscent and two-seeded (Zomlefer 1989). The ellipsoid-cylindric seeds (Wunderlin *et al.* 2021) bear stiff hairs and two elaiosomes, aril-like outgrowths at the micropyle that attract ants that disperse the above-ground seeds (Zomlefer 1989; Weekley and Brothers 2006). The seeds produced by CL flowers are generally larger but much less numerous than those produced by CH flowers (Koontz *et al.* 2017).

*Polygala lewtonii* is endemic to six counties in central peninsular Florida (Brevard, Highlands, Lake, Marion, Polk, and Osceola counties; Wunderlin *et al.* 2021), occurring only in sandhill and scrub habitats on the Lake Wales and the Mount Dora Ridges (Menges *et al.* 2007). These xeric ecosystems experience fire as their dominant mode of ecological disturbance (Menges 2007), with more intense fires in scrub and more frequent fires in sandhill. In scrub, fire increases bare ground, decreases canopy cover (Menges and Hawkes 1998) and increases the frequency and abundance of scrub herbs because of post-burn reductions in shrubs, litter and lichens (Weekley and Menges 2003). Fire positively effects populations of rare, endemic scrub plants by increasing flowering and seedling recruitment (Slapcinsky *et al.* 2010). In sandhill, fire promotes species diversity and temporarily reduces cover (Heuberger and Putz 2003; Reinhart and Menges 2004). Frequent fires in sandhill can increase the cover of grasses and reduce shrub cover (Reinhart and Menges 2004). These xeric uplands in central Florida have suffered from habitat loss and fragmentation (Weekley *et al.* 2008), as well as anthropogenic alteration of disturbance regimes, including longer fire-return intervals and limited use of prescribed fires in land management (USFWS 1999). Fire suppression is one of the most common and serious threats to both sandhill and scrub (Menges 2007). Notably, *P. lewtonii* is fire-maintained and shows population declines with increasing time since fire (Weekley and Menges 2012).

### Sample collection

**Figure 1.**
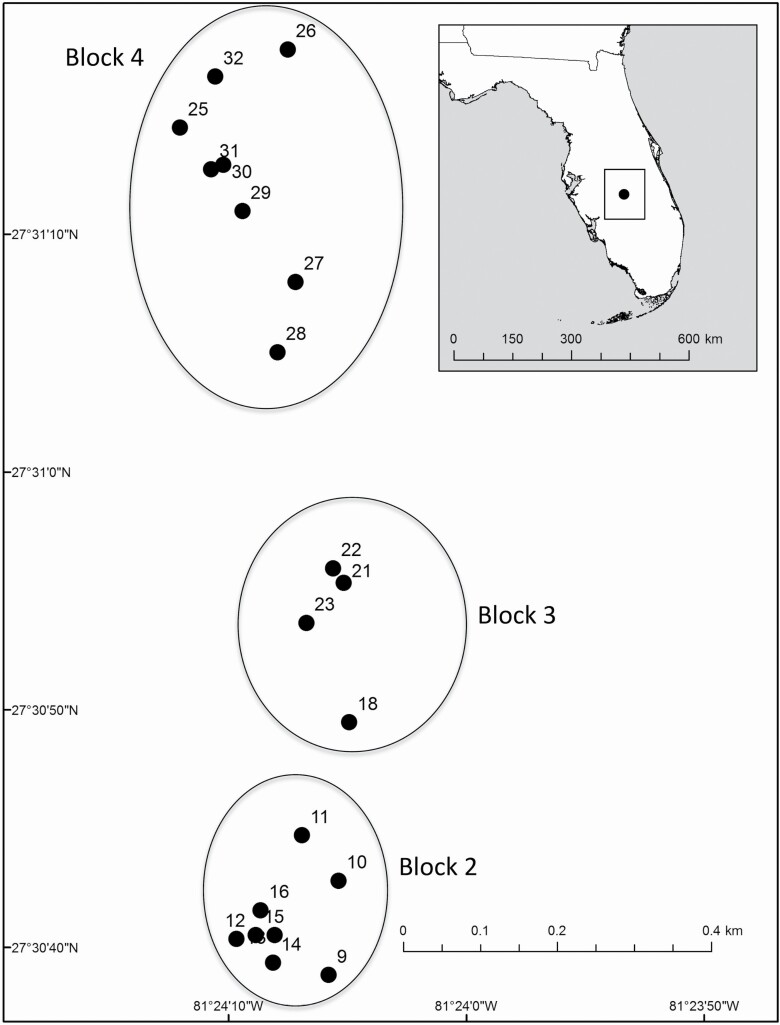
Plots (points) and blocks (circled) at Carter Creek Lake Wales Ridge National Wildlife Refuge in Central Florida. Only plots that were burned and re-sampled are shown.

To understand the outcrossing rate in CH flowers, 5 seeds per plant were collected from the open-pollinated flowers of 10 maternal plants at the Carter Creek Tract of the Lake Wales Ridge Wildlife and Environmental Area (LWRWEA) in the spring of 2017 (see Fig. 1). We also sampled leaf tissue from the maternal plants for comparison with seed samples. To avoid oversampling in a single locality, these individuals were sampled outside the study plots used to understand the dynamics of recolonization after fire (see below). Seeds were stored in paper envelopes and leaf tissue was stored in silica gel desiccant until DNA extraction.

To understand the dynamics of recolonization after fire, individuals were initially sampled from the Carter Creek Tract of the LWRWEA in 2014, as described in Swift *et al.* (2016). The study area was defined in 2014 by compiling all known locations (GPS points) for *P. lewtonii* within the site, yielding an estimated area of ~0.28 km^2^. Within this study area, four blocks were created, and within each block, eight collection plots were established that ranged from 1 to 4 m in radius containing a minimum of nine *P. lewtonii* individuals, with a minimum of 10 m separating plots in the same block. The closest plots in neighbouring blocks were separated by larger distances (mean = 350 m, range 140–630 m between neighbouring blocks). By design, plots were placed at a very fine spatial scale to understand how genotypes and genetic clusters were distributed spatially, with the goal of providing insight into the patterns of reproduction and the dispersal of selfed versus outcrossed seeds. In the 2014 sampling, leaves were collected from nine individuals per plot in each of eight plots per block, totalling 72 individuals per block, or 288 individuals total across the four blocks. A prescribed fire the following year (2 May 2015) burned and killed all plants in the blocks and plots except block 1 (plots 1–8 of Swift *et al.* 2016) and plots 17, 19, 20 and 24. In spring of 2017 (the first season the new germinants flowered), we re-sampled the 20 fully burned plots, collecting leaf tissue from individuals germinating after the fire (Table 1). After fire, we increased sampling to 12 individuals per plot, totalling 240 individuals (Table 1; Fig. 1). To ensure that the pre- and post-fire data sets were comparable, only the pre-fire data from the 20 plots that were completely burned in blocks 2–4 (Fig. 1) were included in the present study, such that the pre-fire data set contained 180 individuals.

**Table 1.** The block name, collection location (plot), year and latitude/longitude for pre- and post-fire sampling of *Polygala lewtonii.* Latitude/longitude has been truncated to three decimal places to protect the plant populations.

Collection locality	Plots included	Block abbreviation	Collection year	Lat, long
Carter Creek-Block 2	9–16	CC2-Pre	2014	27.510, −81.401
		CC2-Post	2017	
Carter Creek-Block 3	18, 21–23	CC3-Pre	2014	27.513, −81.401
		CC3-Post	2017	
Carter Creek-Block 4	25–32	CC4-Pre	2014	27.520, −81.403
		CC4-Post	2017	

### Microsatellite analysis

DNA extractions were performed using a CTAB DNA Extraction Protocol (Doyle and Doyle 1987) modified by using smaller volumes, a smaller initial sample of plant tissue and an additional wash step with cold 95 % ethanol. The seed and leaf tissue samples were genotyped using 11 microsatellite loci as described in Swift *et al.* (2016). Individuals with >50 % missing data were removed from the analysis, resulting in the removal of 10 % of the individuals in the seed data set and ~2 % of individuals in the pre- and post-fire analysis.

### Data analysis of seed samples and simulation of mating system

The seed genotype data were compared to that of the maternal tissue to identify genotyping error. If the seed contained no alleles from the maternal plant at a locus, it was removed from the analysis (see Results section). To analyse whether seeds produced by CH flowers were derived from inbreeding versus outcrossing, we counted the number of non-maternal alleles in the offspring, which were presumably contributed by the pollen donor. Seeds were placed into three categories: individuals having zero non-maternal alleles, those having only one non-maternal allele and those having more than one non-maternal allele. Individuals with zero non-maternal alleles were products of either selfing or biparental inbreeding involving highly genetically similar individuals. Individuals with one non-maternal allele were either the product of cross-pollination between close relatives or genotyping error. Individuals with multiple non-maternal alleles were likely products of outcrossing.

**Figure 2.**
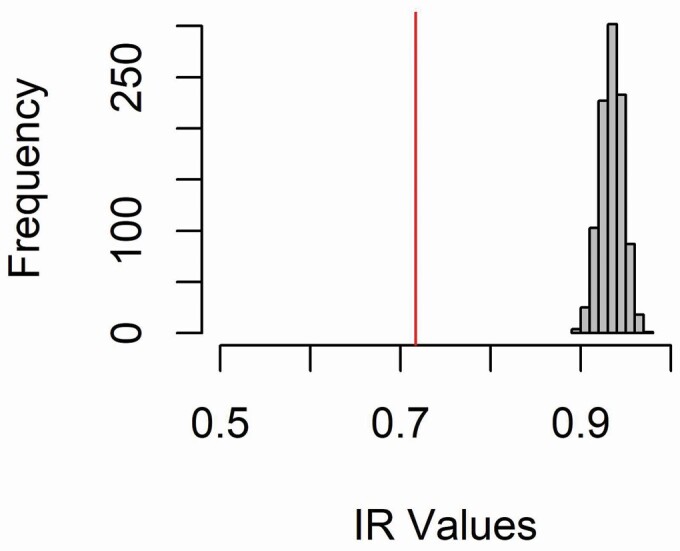
Observed values of heterozygosity in the seeds of CH flowers differ significantly from complete selfing simulations. Distribution of heterozygosity values measured by IR under simulated complete selfing (grey distribution) versus the observed range of heterozygosity in seed samples (red line).

For seed data, we compared the heterozygosity of seeds from CH flowers to that expected under pure selfing by conducting a simulation of pure inbreeding in R version 3.4.1 (R Core Team 2013). For each parent plant, we randomly sampled 10 alleles from each locus that we assembled into five multilocus selfed-seed genotypes. We then repeated the simulation of pure selfing 1000 times. For each simulated seed data set, we calculated five different indices of individual heterozygosity using the function GENHET (Coulon 2010), including PHt, the proportion of heterozygous loci; Hs-observed and Hs-expected, which are standardized measures of observed and expected heterozygosity; IR, or internal relatedness, which weighs shared rare alleles more highly than shared common alleles; and HL, homozygosity by locus, which is expected to follow the opposite pattern of the other four metrics. The script used for this analysis is presented in **Supporting Information—Appendix S1**. We identified significant evidence for outcrossing when the heterozygosity observed in the seed data set fell outside the 95 % quantile of metrics calculated for data sets simulating pure inbreeding (Fig. 2).

### Analysis of changes in genetic diversity and inbreeding after fire

For each block both pre- and post-fire, we used GenAlEx version 6.503 (Peakall and Smouse 2012) to calculate the average number of alleles per locus (*A*) and number of private alleles per population (*A*_P_). We used FSTAT v. 2.94 (Goudet 2002) to calculate allelic richness using rarefaction. We also placed individuals into groups containing all pre-fire or post-fire individuals and used GenAlEx to calculate the number of private alleles; emergence of new private alleles after fire reflects seed bank contributions by individuals not sampled during the original survey. We used GenAlEx to calculate observed and expected heterozygosity (*H*_O_ and *H*_E_); if *H*_O_ increased post-fire, it would indicate that a greater proportion of outcrossed seeds from CH flowers preferentially germinated in response to fire. If *H*_O_ decreased or remained at pre-fire levels (the species previously showed very low *H*_O_ values), it would indicate that seeds that germinated were predominantly produced by selfing or biparental inbreeding.

To detect whether fire stimulated germination of selfed versus outcrossed seeds, we also used GenAlEx to measure the inbreeding coefficient (*F*). Because technical artefacts such as genotyping errors and null alleles (i.e. undetected alleles resulting from amplification failure during polymerase chain reactions) inflate inbreeding coefficients, we used INEST version 2 (Chybicki and Burczyk 2009) to simultaneously measure the frequency of null alleles at each locus and calculate an alternative inbreeding coefficient for each population that takes null alleles and genotyping errors into account (*F*^B^). We used the Bayesian Markov chain Monte Carlo (MCMC) approach with 5 000 000 iterations, a burn-in of 500 000, sampling every 250th iteration. If *F* and *F*^B^ increased post-fire, this would indicate that fire stimulated the germination of seeds produced by inbreeding, whereas a decrease would indicate that a greater proportion of outcrossed seed germinated post-fire.

### Analysis of changes in genetic structure before and after fire

To understand how hierarchical partitioning of genetic variation within and among populations changed in response to fire, we conducted analyses of molecular variance (AMOVA) analyses as implemented in GenAlEx version 6.5 (Excoffier and Lischer 2010). Analyses were conducted separately for pre-fire and post-fire samples, with samples divided into plots nested within blocks. If below-ground seeds predominantly germinated after fire, then we would expect increased among-block and among-plot variance after a fire and decreased within-plot variance, because below-ground seeds are obligately selfing and can disperse only across the length of a rhizome (~1 m). If above-ground seed preferentially germinated, then we would expect among-block and among-plot variation to decrease and within-plot variation to increase because of migration among groups (through both cross-pollination and seed dispersal). This assumes that the 100 % selfed seeds arising from above-ground CL flowers contribute only a small proportion of the total above-ground seed set and that some outcrossing occurs in CH flowers.

To estimate genetic structure independently of *a priori* population designations, we utilized InStruct version 1.0 (Gao *et al.* 2007), which was specifically designed to measure genetic structure in highly inbreeding species. It differs from STRUCTURE (Pritchard *et al.* 2000) by eliminating the assumption of Hardy–Weinberg equilibrium within genetic clusters and jointly estimating the selfing rate and population structure. We analysed a data set composed of both the pre- and post-fire data using the default settings in InStruct, except we varied the number of groups, *K*, from 1 to 15, employed 10 independent chains of the MCMC algorithm for each *K*, used a burn-in of 500 000 iterations and a run length of 1 000 000 iterations for each chain, following the recommendations of Gilbert *et al.* (2012). To ensure convergence and repeatability, we examined the groupings across all runs at each *K* in CLUMPAK (Kopelman *et al.* 2015). To determine the optimal value of *K*, we plotted the Deviance Information Criterion (DIC) and the −ln likelihood values from InStruct and selected the value where they plateaued and showed clear patterns of genetic structure.

To quantify how admixture and genetic structure changed after a fire, we identified the predominant genetic cluster to which individuals were assigned, then calculated the percentage of individuals in each plot both pre- and post-fire that showed their greatest assignment to the predominant cluster. We also calculated average admixture proportions at the predominant InStruct cluster across all individuals in a plot, both pre- and post-fire. If below-ground selfed seed predominantly germinated after a fire, then we would expect post-fire plots to have more individuals and greater average admixture proportions assigned to the predominant genetic cluster. If above-ground seeds preferentially germinated after a fire, then because these seeds are capable of dispersal, we would expect post-fire plots to show fewer individuals assigned to a predominant genetic cluster. Provided that above-ground seeds showed some outcrossing, we would also expect lower average admixture proportions assigned to the predominant genetic cluster.

Finally, we repeated analyses described above with individuals grouped into the predominant genetic cluster assigned by INSTRUCT. Analyses included estimates of genetic diversity, AMOVA and average admixture proportions at the majority cluster.

## Results

### Outcrossing rate in CH flowers

Seeds produced by CH flowers showed evidence of limited outcrossing. After removing 10 seeds from the analysis because of genotyping failure or genotyping errors, the final data set contained 40 seeds from nine maternal individuals, of which 30 out of 40 (75 %) had only maternal alleles present, 4 of 40 (10 %) differed from their mother by only one allele and 6 of 40 (15 %) differed from their mother by three or more alleles. When comparing observed inbreeding metrics to those simulated under 100 % selfing, the IR among seeds from open-pollinated flowers (0.718) was well below the range of values simulated for pure selfing (0.900–0.985; Fig. 2). Heterozygosity (PHt, HL, Hs-observed and Hs-expected) was significantly outside the range expected under simulated selfing **[see****Supporting Information—Table S1****]**.

### Genetic diversity before and after fire

Burning had modest effects on genetic diversity. The mean number of alleles per population and allelic richness showed slight increases after fire (mean *A*_Pre_ = 3.00, mean *A*_Post_ = 3.303; mean *A*_R-Pre_ = 2.71, mean *A*_R-Post_ = 2.79; **see****Supporting Information—Table S2**). When grouped into blocks, the mean number of private alleles per block was 3.0 pre-fire and was 1.33 post-fire. When comparing private alleles between all pre-fire versus all post-fire samples, seven alleles were unique pre-fire and eight were unique post-fire **[see****Supporting Information—Table S1****]**. All eight post-fire private alleles were not observed in the previous range-wide study (Swift *et al.* 2016). All private alleles were present at low frequencies (range = 0.004–0.031 %, mean = 0.01 %; **see****Supporting Information—Table S2**), but together, alleles unique to one sampling time accounted for ~17 % of the total alleles.

Expected heterozygosity (*H*_E_), observed heterozygosity (*H*_O_) and inbreeding coefficients increased slightly after fire. The mean pre-fire *H*_E_ averaged across loci was 0.311, whereas it was 0.322 post-fire (Table 2). Values of *H*_O_ also increased somewhat after burning (pre-fire mean = 0.039, post-fire mean = 0.047; Table 2). As found previously, all *H*_O_ values were much lower than *H*_E_, such that inbreeding coefficients (*F*) reached values close to one. Inbreeding coefficients increased slightly from pre-fire values (Table 2) from a mean of 0.795 in pre-fire populations to a mean of 0.811 post-fire (Table 2). After correction for null alleles using INEST, *F*^B^ remained high and increased from a mean of 0.815 in pre-fire blocks to a mean of 0.845 in post-fire blocks (Table 2).

**Table 2.** The effects of fire on genetic diversity in *Polygala lewtonii*. Parameters include *N*, number of samples, *H*_O_, observed heterozygosity, *H*_E_, expected heterozygosity, *A*, average number of alleles per locus, *A*_R_ allelic richness based on a minimum sample size of 21 individuals, *A*_*P*_, number of private alleles, *F*, inbreeding coefficient and *F*^B^, the inbreeding coefficient taking null alleles into account. See Table 1 for block abbreviations.

Block	N	*H*_O_	*H*_E_	*A*	*A*_R_	*A*_P_	*F*	*F* ^B^
CC2-Pre	68	0.06	0.33	3.09	2.72	2	0.80	0.74
CC3-Pre	33	0.02	0.35	2.55	2.61	1	0.93	0.90
CC4-Pre	70	0.03	0.25	3.36	2.80	4	0.66	0.81
Mean-Pre		0.04	0.31	3.00	2.71	3	0.79	0.81
CC2-Post	96	0.08	0.34	3.73	2.61	3	0.73	0.77
CC3-Post	48	0.03	0.37	2.91	2.99	1	0.95	0.93
CC4-Post	96	0.04	0.26	3.27	2.77	0	0.76	0.85
Mean-Post		0.05	0.32	3.30	2.79	1.33	0.81	0.85

### Patterns of genetic structure among blocks before and after fire

Burning changed the hierarchical partitioning of genetic variation, with greater variation partitioned among blocks and plots and less variation found among individuals within plots after fire. Pre-fire samples had 22 % of the variation partitioned among blocks, 28 % among plots, 43 % among individuals within plots and 7 % within individuals (Table 3). Post-fire, 28 % of the variation was found among blocks, 34 % among plots, 28 % was found among individuals within plots and 10 % within individuals (Table 3). *F*-statistics derived from AMOVA show a pattern of increased *F*_RT_, *F*_SR_ and *F*_ST_ and decreased *F*_IS_ and *F*_IT_ after fire (Table 3).

**Table 3.** The effects of fire on the hierarchical partitioning of genetic variation in *Polygala lewtonii* using AMOVA analysis. Analyses were conducted before and after the 2015 fire, with individuals grouped by plots nested within blocks.

	Degrees of freedom	Sums of squares	Estimated variance	Mean squares	Percent variation	*F*-statistics
Pre-fire						
Among blocks	2	184.503	92.252	0.682	22 %	*F*_RT_ = 0.219
Among plots	17	305.387	17.964	0.880	28 %	*F*_SR_ = 0.361
Among individuals	151	438.920	2.907	1.351	43 %	*F*_ST_ = 0.501
Within individuals	171	35.000	0.205	0.205	7 %	*F*_IS_ = 0.868
Total	341	963.810		3.119	100 %	*F*_IT_ = 0.934
Post-fire						
Among blocks	2	282.939	141.469	0.763	28 %	*F*_RT_ = 0.277
Among plots	17	413.922	24.348	0.939	34 %	*F*_SR_ = 0.472
Among individuals	220	400.917	1.822	0.771	28 %	*F*_ST_ = 0.618
Within individuals	240	67.500	0.281	0.281	10 %	*F*_IS_ = 0.733
Total	479	1165.277		2.753	100 %	*F*_IT_ = 0.898

**Figure 3.**
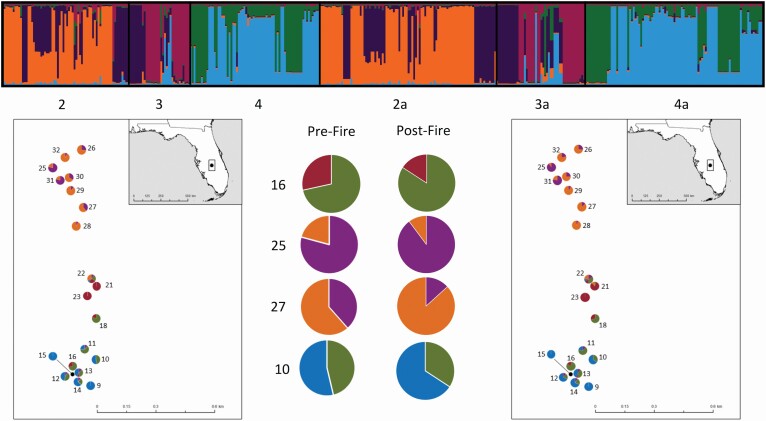
Patterns of genetic structure from pre- (blocks 2, 3 and 4) and post-fire (blocks 2a, 3a and 4a) populations show a decline in admixture as a result of fire. InStruct graph and maps show population structure for pre- and post-fire, and corresponding pie charts. These cluster assignment proportions are also shown as pie charts superimposed on their respective sampling plots. The pie charts in the centre (plots 16, 25, 27 and 10) are highlighted as they show examples of reduced post-fire admixture.

InStruct analyses revealed increased genetic structure and decreased admixture after burning. The DIC and −ln likelihood values plateaued between *K* = 4 and 6 **[see****Supporting Information—Fig. S1****]**, but we present *K* = 5 in Fig. 3 because it had the clearest assignment to clusters and was consistent with the results of Swift *et al.* (2016). Generally, more individuals were assigned to the most common genetic cluster in each plot or block, and individuals showed greater average assignments to the predominant cluster, indicating less admixture post-fire (Table 4). In 8 of 20 total plots (such as plots 15, 18, 25 and 28; Fig. 3), the number of individuals assigned to the most common InStruct cluster increased post-fire, whereas cluster assignment remained at 100 % for eight plots, and decreased in only two plots. The proportion of admixture accounted for by the majority cluster also increased in all but two plots, indicating individuals were less admixed between InStruct clusters (Table 4). These results indicate that genetically similar individuals were more tightly spatially clustered after a fire and showed less admixture among plots.

**Table 4.** The effects of fire on genetic structure and admixture in *Polygala lewtonii.* Results show the proportion of individuals assigned to a majority cluster in InStruct pre- and post-fire, and the average admixture proportions of assignment to the predominant cluster in plots before and after fire.

Plot	Block	Pre-fire % individuals assigned to predominant cluster	Post-fire % individuals assigned to predominant cluster	Pre-fire average admixture proportion for predominant cluster	Post-fire average admixture proportion for predominant cluster
9	2	100 %	100 %	0.904	0.941
10	2	65.5 %	66.7 %	0.503	0.624
11	2	100 %	100 %	0.593	0.637
12	2	62.5 %	91.7 %	0.466	0.655
13	2	66.7 %	58.3 %	0.426	0.434
14	2	75.0 %	83.3 %	0.592	0.665
15	2	100 %	100 %	0.916	0.962
16	2	88.9 %	100 %	0.669	0.773
18	3	100 %	100 %	0.746	0.776
21	3	100 %	83.3 %	0.952	0.802
22	3	42.9 %	50 %	0.326	0.352
23	3	100 %	100 %	0.920	0.951
25	4	77.8 %	100 %	0.764	0.863
26	4	62.5 %	66.7 %	0.713	0.708
27	4	66.7 %	100 %	0.576	0.829
28	4	100 %	100 %	0.875	0.903
29	4	100 %	100 %	0.862	0.868
30	4	62.5 %	75 %	0.649	0.706
31	4	100 %	100 %	0.774	0.761
32	4	100 %	100 %	0.877	0.749
Average across plots		84 %	89 %	0.705	0.748

When individuals were grouped by InStruct cluster, the changes in genetic diversity were consistent with those in which individuals were grouped by block or plot. We observed minor changes in genetic diversity after fire **[see****Supporting Information—Table S3****]**, an increase in among-group genetic variation and a decrease in within-group and within-individual variation in AMOVA analyses **[see****Supporting Information—Table S4****]**, and a decrease in admixture in InStruct **[see****Supporting Information—Table S5****]**.

## Discussion

The goal of this study was to test the hypothesis that one role of the obligately selfing below-ground seeds of an amphicarpic species is to recolonize a site after disturbance (Cheplick and Quinn 1987, 1988), or alternatively, that fire promotes the germination of above-ground seeds (Swift *et al.* 2016). We tested these hypotheses by analysing how genetic diversity, inbreeding and genetic structure changed after fire in the amphicarpic species, *P. lewtonii*.

Because we would only be able to detect differential germination post-fire if the outcrossing rate differs among above-ground and below-ground flowers, we first quantified outcrossing rates in CH flowers of *P. lewtonii*. Overall, 30 of 40 individuals (75 %) had only maternal alleles present, indicative of self-fertilization, biparental inbreeding or geitonogamy. Given the low seed set previously observed in *P. lewtonii* when pollinators were excluded (Weekley and Brothers 2006), the latter two may be more plausible mechanisms for inbreeding in CH flowers. The high inbreeding rate in CH flowers also helps explain the low observed heterozygosity and high inbreeding coefficients previously observed in *P. lewtonii* (Swift *et al.* 2016). Although Swift *et al.* (2016) proposed that the high inbreeding rate could be due to unsuitable environmental conditions causing poor germination of outcrossed seed, these results indicate that *P. lewtonii* shows high inbreeding simply because 75 % of the seeds in CH flowers are produced through inbreeding, in addition to the 100 % inbred seeds set by CL flowers. The high inbreeding rate in CH flowers may instead be due to poor pollinator efficiency (Swift *et al.* 2016), the effects of which may be magnified by the high relatedness of neighbouring individuals, especially those emerging from underground CL seeds.

The remaining 25 % of seeds from CH flowers contained at least one non-maternal allele, indicating reproduction via cross-pollination. Even though the cross-pollination rate was low in CH flowers, simulations showed that it was significantly greater than that of fully selfing flowers. Although the total above-ground seed set is derived from both the CH and CL flowers, because CH flowers produce ~13-fold more seeds than above-ground CL flowers, the above-ground CL flowers likely contribute little to the overall selfing rates of above-ground seed. Thus, the total outcrossing rate for all seed produced above-ground (from both CL and CH flowers) would still be greater than that expected for the 100 % selfed below-ground seed, indicating that it would be possible to detect whether above-ground or below-ground seed predominantly germinated after a fire.

Pre- and post-fire comparisons of genetic diversity, inbreeding coefficients and genetic structure were most consistent with greater post-fire germination of seeds produced by inbreeding. Pre-fire observed heterozygosity levels were very low, and were much smaller than expected heterozygosity, resulting in large positive inbreeding coefficients (*F* and *F*^B^), indicating high rates of selfing or inbreeding. After fire, both observed and expected heterozygosity increased slightly, likely due to a slight increase in allelic diversity post-fire reflecting new alleles emerging from the seed bank. However, post-fire expected heterozygosity increased slightly more than observed heterozygosity, resulting in greater inbreeding coefficients. If seeds produced by above-ground flowers preferentially germinated after a fire, then given the greater seed set of CH flowers (Koontz *et al.* 2017) and the estimates of outcrossing obtained in the first experiment, we undoubtedly would have observed a post-fire decrease in inbreeding coefficients. The increased inbreeding suggests that the species may rely more strongly on selfed seed in post-fire recolonization.

Genetic structure also increased after fire, which is consistent with the reduced dispersal of below-ground CL seeds. Following fire, AMOVA analyses showed an increase in the proportion of genetic variation attributable to allele frequency differences among *a priori* population groups at both the block and plot levels. This pattern was consistent with InStruct results, which showed that genetically similar individuals were more tightly spatially clustered and that individuals were less admixed after a fire. Some factors that increase genetic structure are inbreeding between geographically proximal relatives and reduced pollination distances (Loveless and Hamrick 1984). In particular, self-fertilization eliminates pollen movement, reduces effective population sizes and results in strong genetic drift, thereby resulting in strong genetic structure among groups (Nordborg and Donnelly 1997; Wright *et al.* 2013; Hartfield *et al.* 2017). Greater among-group variation is also expected when seeds have limited dispersal capability (Hamrick *et al.* 1993). The changes in the hierarchical partitioning of genetic variation are therefore consistent with an increased post-fire contribution by seeds that dispersed only short distances, a characteristic of below-ground CL flowers. An association between fire and increased production of seed from CL flowers was also found in a recent study of *Cologania broussonetii*, a perennial re-sprouting herb with both CH and CL flowers, that found that expression of CL flowers was maximized under frequent fires (Carbone *et al.* 2021).

However, contrary to the results of other analyses of inbreeding (*F* and *F*^B^), the AMOVA-derived *F*_IS_ decreased after fire. The apparent discrepancy likely reflects differences in how different methods quantify the effects of genetic drift on inbreeding. GenAlex and INest measure inbreeding based on the probability of identity by descent (i.e. inbreeding effective population sizes; Chybicki and Burczyk 2009; Peakall and Smouse 2012), whereas AMOVA measures inbreeding based on changes in the variance in allele frequencies (i.e. variance effective population sizes; Weir and Cockerham 1984; Excoffier *et al.* 1992). These metrics exhibit different responses to both inbreeding and population size fluctuations (Templeton 2006) that are relevant to how fire influences *P. lewtonii*. In partially selfing populations, such as this one, inbreeding effective population sizes change less with the number of individuals than do variance effective population sizes (Li 1965). Therefore, changes in *F* and *F*^B^ probably represent a change in the mating system due to an increase in the proportion of selfed seeds, whereas *F*_IS_ also depends on how many plants reproduce. Furthermore, when population sizes fluctuate, the accumulation of inbreeding tends to reflect the parental generation, whereas the variance in allele frequencies tends to reflect variation in the offspring generation (Crow and Kimura 1970). In this system, increases in *F* and *F*^B^ likely reflect increased reproductive success of parents that produced below-ground seed by selfing before the fire, whereas the decrease in *F*_IS_ probably reflects the larger pool of more variable offspring that recruited from the persistent seed bank.

Although the current study is among the first to show that seeds produced by below-ground flowers in an amphicarpic species were important in post-fire colonization, the exact mechanism underlying this phenomenon is unknown. Amphicarpy is hypothesized to be an adaptation to ecological disturbance (Symonides 1988). For amphicarpic species that inhabit fire-maintained ecosystems, below-ground seeds may be over-represented in the seed bank from which the plant population germinates after fire because they occur deeper in the soil and may be less likely to be damaged by fire (Zhang *et al.* 2020). This may particularly be the case when fires occur during the period of seed production by CH flowers, which was the case in the present study. The possibility that fire may damage above-ground seeds also helps explain why we did not find an increase in outcrossing after a fire, even though CH flowers produce much greater quantities of seed than either type of CL flowers (Koontz *et al.* 2017). Another possible explanation for why the seeds produced below-ground showed increased contributions to the population after a fire is because they are larger and experienced more favourable ecological conditions for germination than those produced above-ground (e.g. greater moisture below the soil surface), which is supported by previous studies that found that seeds of one amphicarpic grass species buried below-ground often showed greater germination and survival after a fire (Cheplick and Quinn 1987, 1988).

These results support a cyclical pattern to the reproduction, genetic diversity and genetic structure in *P. lewtonii*, which appears to be an adaptation to a fire-maintained ecosystem. After a fire, the seeds from below-ground CL flowers likely predominantly recolonize a site, resulting in high inbreeding coefficients, low admixture and strong patterns of genetic structure. In years that do not experience fire, more seedlings may arise from seeds produced by the more prolific CH flowers (Koontz *et al.* 2017). Although we found that only 25 % of the reproduction achieved by CH flowers was from outcrossing, several years of reproduction primarily arising from seeds produced by CH flowers would lead to a gradual increase in heterozygosity and a decrease in inbreeding coefficients and population structure, as found in the pre-fire data set. The genetic variation generated by outcrossing in CH flowers may help facilitate adaptation of *P. lewtonii* to environmental change (Oakley *et al.* 2007). Although many studies have debated the stability of mixed mating systems (Holsinger 1991; Goodwillie *et al.* 2005), the mixed mating system and associated amphicarpy in *P. lewtonii* and other amphicarpic species may be stable because it provides one potential strategy to produce seeds that can germinate in the challenging conditions found in disturbance-prone environments (Hidalgo *et al.* 2016; Zhang *et al.* 2020).

We also observed a clear shift in alleles present in the population following the fire event, which provides some indication of seed longevity in the soil seed bank. Although some low-frequency alleles were lost after fire, a roughly equal number of new alleles emerged at low frequencies post-fire. None of the alleles that emerged after fire were encountered in previous range-wide population genetic analysis of the species (Swift *et al.* 2016). Because this population was completely burned, seed dispersal is limited, and these alleles were not detected previously, it is likely that at least some of them were preserved in the soil seed bank and re-emerged after fire. The first sampling occurred in 2014 and new alleles appeared in 2017, indicating that seeds remained viable in the seed bank at least 4 years, but possibly longer, before germination was stimulated by fire. Given that the success of the amphicarpic life history strategy depends on seeds maintaining viability in the soil, this provides further support for the hypothesis of post-disturbance recolonization by below-ground seeds. Seed banks may serve as a buffer against the negative genetic effects of disturbance in fire-prone ecosystems (Dolan *et al.* 2008), and new alleles emerging from the seed bank may buffer the population from a loss of genetic diversity after disturbance.

In addition to being an integral part of the natural cycle of disturbance and playing an important role in maintaining sandhill and scrub ecosystems (USFWS 1999; Menges 2007), fire has multiple positive effects on *P. lewtonii*, including increased recruitment and survival of post-burn recruits (Weekley and Menges 2012). This study highlights *P. lewtonii*’s highly specific adaptations that allow it to thrive in response to fire, underscoring the importance of using fire management to sustain populations of this endangered species (Weekley and Menges 2012). More generally, the results support previous hypotheses about the functional significance of amphicarpy as an adaptation to disturbance-prone environments. Amphicarpy has evolved independently multiple times in species occupying fire-maintained ecosystems (Zhang *et al.* 2020), emphasizing the importance of fire as a selective force shaping the evolution of plants and the importance of maintaining historical fire regimes to ensure the conservation of biodiversity.

## Supporting Information

The following additional information is available in the online version of this article—

**Table S1.** Measures of individual heterozygosity (PHt, Hs-observed and Hs-expected, internal relatedness [IR] and homozygosity by locus [HL]) from genotyped seeds of *Polygala lewtonii* and simulated selfing.

**Table S2.** Alleles unique to either the pre- or post-fire sample of *Polygala lewtonii*.

**Table S3.** Genetic diversity in pre- and post-fire populations of *Polygala lewtonii* grouped by InStruct cluster.

**Table S4.** Results of analyses of the partitioning of genetic variation in *Polygala lewtonii* using AMOVA with individuals grouped by InStruct clusters.

**Table S5.** The average admixture proportions of *Polygala lewtonii* individuals in InStruct clusters.

**Figure S1.** Plots of Deviance Information Criterion (DIC) and ln likelihood (ln(*K*)) curves for InStruct analysis of *Polygala lewtonii* as presented in Fig. 3 at values of *K* = 1–10.

**Appendix S1.** Code for inbreeding simulation.

plab067_suppl_Supplementary_Material

## Data Availability

Microsatellite data available via FigShare at https://doi.org/10.6084/m9.figshare.16713214.

## Notes

**Populations & Communities. Chief Editor**: Jean Burns
